# Dynamic Alginate Hydrogel as an Antioxidative Bioink for Bioprinting

**DOI:** 10.3390/gels9040312

**Published:** 2023-04-07

**Authors:** Wenhai Zhang, Mitchell Kuss, Yi Yan, Wen Shi

**Affiliations:** 1 Orthopedic Department, Tianjin Hospital, Tianjin 300211, China; 2 Mary & Dick Holland Regenerative Medicine Program, University of Nebraska Medical Center, Omaha, NE 68198, USA; 3 Healthcare Security Office, Union Hospital, Tongji Medical College, Huazhong University of Science and Technology, Wuhan 430074, China

**Keywords:** dynamic hydrogel, 3D bioprinting, antioxidative, boronate ester

## Abstract

3D bioprinting holds great potential for use in tissue engineering to treat degenerative joint disorders, such as osteoarthritis. However, there is a lack of multifunctional bioinks that can not only support cell growth and differentiation, but also offer protection to cells against injuries caused by the elevated oxidative stress; this conditions is a common characteristic of the microenvironment of the osteoarthritis disease. To mitigate oxidative stress-induced cellular phenotype change and malfunction, an anti-oxidative bioink derived from an alginate dynamic hydrogel was developed in this study. The alginate dynamic hydrogel gelated quickly via the dynamic covalent bond between the phenylboronic acid modified alginate (Alg-PBA) and poly (vinyl alcohol) (PVA). It presented good self-healing and shear-thinning abilities because of the dynamic feature. The dynamic hydrogel supported long-term growth of mouse fibroblasts after stabilization with a secondary ionic crosslinking between introduced calcium ions and the carboxylate group in the alginate backbone. In addition, the dynamic hydrogel showed good printability, resulting in the fabrication of scaffolds with cylindrical and grid structures with good structural fidelity. Encapsulated mouse chondrocytes maintained high viability for at least 7 days in the bioprinted hydrogel after ionic crosslinking. Most importantly, in vitro studies implied that the bioprinted scaffold could reduce the intracellular oxidative stress for embedded chondrocytes under H_2_O_2_ exposure; it could also protect the chondrocytes from H_2_O_2_-induced downregulation of extracellular matrix (ECM) relevant anabolic genes (ACAN and COL2) and upregulation of a catabolic gene (MMP13). In summary, the results suggest that the dynamic alginate hydrogel can be applied as a versatile bioink for the fabrication of 3D bioprinted scaffolds with an innate antioxidative ability; this technique is expected to improve the regenerative efficacy of cartilage tissues for the treatment of joint disorders.

## 1. Introduction

The degeneration of cartilage and other joint tissues can cause great pain and disability to the patients; it is also a big economic burden to the healthcare system [1]. Current approaches for treating degenerative joint diseases, such as osteoarthritis, are very limited and not satisfactory [2]. The biofabrication technique of 3D bioprinting, which enables layer-by-layer deposition of cells and biomaterials (the bioink) in a controlled spatial manner, results in the generation of live, functional tissues with complex structures and opens new doors for restoring the degenerated joint tissue [3]. It also allows the fabrication of patient-specific constructs with porous structures that facilitate nutrient and metabolic waste exchange, thereby reducing the de-differentiation of embedded chondrocytes and enhancing the cartilage tissue engineering efficacy [4].

3D bioprinting is heavily dependent on the bioink to mimic the extracellular matrix (ECM) of the tissue microenvironment and support chondrocyte growth and differentiation [5]. Many bioinks for chondrocyte printing have been developed, most of which were derived from natural polymers, such as alginate, gelatin, chitosan, hyaluronic acid, and silk, because of their high biocompatibilities [6]. The alginate bioink is of particular interest because it provides a 3D environment that not only promotes chondrocyte proliferation but also maintains the chondrogenic phenotype [7]. However, the alginate solution alone does not meet the requirement for extrusion-based printing because of the relatively low viscosity of the solution. The addition of other polymers or viscosity modifiers is always required to prepare alginate based bioinks [8]. There is a constant interest in developing novel alginate bioinks to enhance cartilage tissue engineering [9].

Other than the ECM factor, the oxidative stress species (ROS) are also important for chondrocyte function. While ROS at low levels serve as a signaling pathway for normal biological activities, elevated ROS are commonly found in the degenerative joint tissues of patients and are considered as a major contributor to the progression of OA [10,11,12]. The excess ROS can change the cellular phenotype and impair the chondrocyte function by disrupting the balance between anabolism and catabolism of ECM components, leading to continuous damage to the cartilage integrity [13]. It is considered that chondrocytes delivered through 3D bioprinted constructs are still vulnerable to phenotype and function changes after being exposed to the excess ROS in the oxidative microenvironment of the degenerated joints, which may hinder the efficacy of the regeneration.

To attenuate the excess ROS, the antioxidant can be introduced. Many previous studies have indicated that antioxidant therapy showed great potential in arresting the progression of osteoarthritis [14]. Although the antioxidants can be delivered in either free molecule forms or encapsulated in nanoparticles or microparticles, both systems suffer from certain problems if they are incorporated into a bioink. For the free molecule forms, the release of antioxidative small molecules or enzymes from bioink is difficult to control in terms of the initial burst release and quick diffusion [15]. For the nanoparticle and microparticle forms, due to a loading efficiency issue, a large number of cargoes must be loaded to achieve long-term release, which not only increases the system’s complexity but also raises biocompatibility concerns towards the encapsulated cells in the bioink [16]. To resolve these challenges, the development of bioinks with innate antioxidative properties seems to be a better option.

In order to improve the efficacy of the tissue regeneration in ROS relevant diseases, such as stroke and myocadiac infarction, injectable hydrogels with inherent antioxidative properties have emerged as promising platforms for scavenging the exogeneous ROS and enhancing the survival of hydrogel-delivered cells in the oxidative microenvironment of those diseases [17,18,19]. However, most of those hydrogels are not printable. There is a necessity of developing multifunctional bioinks that can not only support the chondrocytes growth but also preserve the phenotype and function of chondrocytes under oxidative stress. We have developed a variety of printable hydrogels with innate antioxidative properties based on boronate ester bond-based dynamic hydrogels [18,20,21,22]. Those dynamic hydrogels were easily formed between the grafted phenylboronic acid (PBA) from the hyaluronic acid (HA) conjugate and the abundant 1,2-diols in the polyvinyl alcohol (PVA) in the aqueous solution under ambient conditions [23]. The additional antioxidative property comes from the H_2_O_2_ scavenging ability of the boronate ester [24]. Moreover, the boronate ester bond provided dynamic hydrogels with good shear-thinning properties and the hydrogels were successfully applied as bioinks to fabricate constructs with good structural fidelity [18,22]. However, scaffolds printed using the boronate ester bond-based hydrogels usually had poor stability due to the relatively weak binding strength between the phenylboronic acid and the diols; therefore, secondary crosslinking approaches are always necessary for in vivo application [25]. A chemical crosslinking strategy was utilized in a previous study by simultaneously modifying the HA molecule with a PBA group; an acrylate group and thiolated gelatin was also introduced as the secondary crosslinking agent to stabilize the printed dynamic hydrogel [22]. This approach was effective but tedious as many chemical reactions and purifications were required to prepare the hydrogel precursors. It will be interesting to develop a dynamic alginate hydrogel that can be easily stabilized through ionic interaction without the use of another polymer crosslinker.

In the current study, a novel bioink derived from boronate ester dynamic covalent bond-based alginate hydrogel with good shear thinning and antioxidative properties was developed; this bioink was further applied for 3D bioprinting in tissue engineering, especially the regeneration of degenerated cartilage tissues. The rheological, self-healing, shear-thinning, and structural properties of the dynamic alginate hydrogel were thoroughly characterized. The degradation of the dynamic hydrogel before and after secondary ionic crosslinking was also evaluated. We then applied the chondrocyte loaded dynamic hydrogel as a bioink and assessed the performance of the printing in terms of the structural fidelity and cell viability. In the end, to specify the value of the antioxidative bioink, an in vitro H_2_O_2_ exposure study was performed to mimic the oxidative stress in degenerated cartilage tissues. Our results indicated the PBA modified alginate is cyto-compatible and multifunctional and can be used in 3D printing at low alginate concentration without any viscosity modifier. The novel bioink could be easily stabilized with ionic crosslinking. Moreover, the novel bioink could help the embedded chondrocytes to keep the cartilage-specific anabolic and catabolic gene expressions under elevated ROS in vitro.

## 2. Results and Discussion

### 2.1. Fabrication and Characterization of Alg-PBA Hydrogels

**Figure 1:**
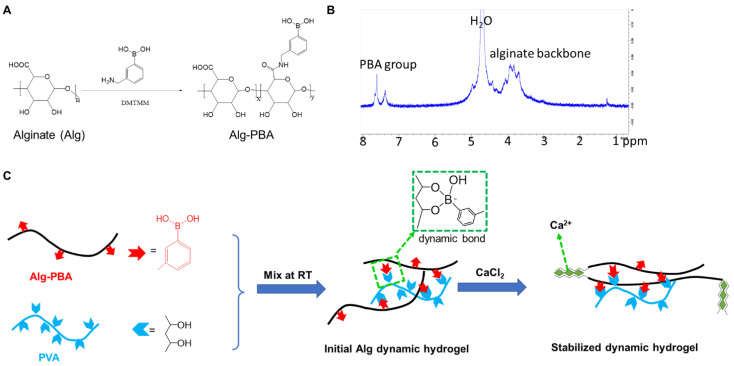
Schematic illustration for preparation of dynamic alginate hydrogel and its stabilization. (**A**) Synthesis strategy of alginate derived with PBA functional group (Alg-PBA); (**B**) ^1^H NMR spectra of Alg-PBA; (**C**) schematic representation of formation of dynamic alginate hydrogel stabilized with calcium ions.

The Alg-PBA conjugate was synthesized by grafting the 3-aminomethyl PBA to the alginate backbone through an amide bond. The schematic synthesis step of Alg-PBA is shown in Figure 1A. DMTMM was used instead of 1-ethyl-3-(3-dimethylamino propyl) carbodiimide (EDC) as the coupling agent as DMTMM showed better conjugation efficiency for carbohydrate modification and fewer impurities after reaction compared to the EDC coupling agent. This could lead to consistency in the conjugate step and lessen the toxicity issue from the reactant residual after the reaction. The ^1^H NMR analysis confirmed the conjugation (Figure 1B) and the grafting ratio was revealed after the inclusion of a reference material DMF following a previous study method [26]. It was found that ~32% of the carboxylic acid in the alginate backbone was amidated by the PBA moiety (Figure S1). It was further confirmed that double the amount of DMTMM and PBA in the conjugation reaction increased the grafting efficiency and resulted in nearly two times PBA moiety grafted in the alginate backbone. Although previous studies have synthesized similar phenyboronic acid derivatized alginate conjugates, most of these studies employed EDC as the coupling agent [27,28]. However, based on our experience, EDC reaction always introduced more residuals that were difficult to remove during the dialysis step. Another disadvantage was that the EDC reaction was never stoichiometric and many optimization reactions or trials were required to achieve the target grafting ratio. Overall, we found that DMTMM was an effective and controllable coupling agent to conjugate the PBA group to the alginate.

The injectable, dynamic hydrogel was prepared by mixing the Alg-PBA solution with the PVA solution at a 3:1 volume ratio. Rapid gelation (<10 s) happened at room temperature after the fast complexation/boronate ester dynamic bond formation between the PBA group from the modified alginate and the diol group in PVA [29]. PVA is commercially available and biologically inert; it has also been safely used for developing cartilage scaffolds [30]. PVA provides a great number of diol groups to form the complex with PBA group, thus leading to hydrogel network formation. PVA has been successfully applied to form bioinks with PBA functionalized hyaluronic acid. However, the boronate ester bond is not stable during cell culture and in vivo. Therefore, we introduced secondary crosslinking enhance the stability, which was formed through the ionic interactions between the divalent calcium ions and the carboxylate groups from the alginate strands; this was a common method to prepare alginate hydrogels (Figure 1C).

**Figure 2:**
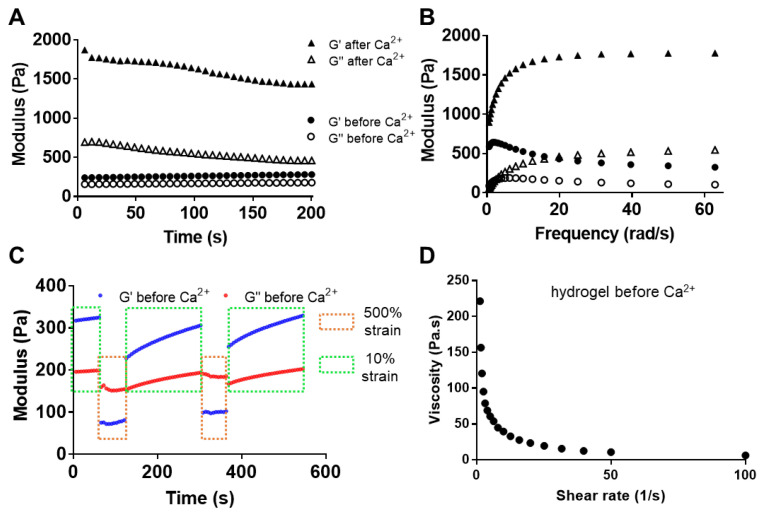
Rheological properties of dynamic alginate hydrogel. (**A**) Time-sweep studies indicating storage modulus (G′) and loss modulus (G″) change in dynamic hydrogels before and after CaCl_2_ crosslinking. (**B**) Frequency-sweep studies indicating G′/G″ of the dynamic Alg-PBA-PVA hydrogel before and after CaCl_2_ crosslinking. (**C**) Recovery of G′ and G″ of dynamic Alg-PBA-PVA hydrogel under alternate step strains of 10% (180 s) and 500% (60 s) for two consecutive cycles (blue line: storage modulus, red line: loss modulus); (**D**) Viscosity of the dynamic hydrogel with increased shear rate from 1 s^−1^ to 100 s^−1^.

The rheological properties of the Alg-PBA hydrogels were then investigated. Although we only focused on one Alg-PBA precursor concentration (2.5%) in this study, our preliminary results indicated the storage modulus (G′) of the Alg-PBA-PVA hydrogels was dependent on the precursor content (data not given). The storage modulus (G′) of the Alg-PBA-PVA hydrogels was 280 ± 20 Pa and was higher than the loss modulus (G″) at 10% strains. In contrast, the G′ of the calcium crosslinked hydrogel reached 1440 ± 40 Pa after equilibrium (Figure 2A). The frequency-sweep tests indicated good elastic characteristic of both hydrogels as the G′ were constantly larger than G″ across the frequency range tested (Figure 2B).

The dynamic boronate ester bond enabled a self-healing property for the dynamic alginate hydrogel, which was first implied by the rheological alternate step strain and recovery test (Figure 2C). The recovery process consisted of alternate step strains of 10% and 500% in each cycle. When the dynamic hydrogel was subjected to a relatively large strain (500%), the hydrogel network collapsed and the G′ value sharply decreased from 315 Pa to 75 Pa. Once the strain returned to the initial 10% strain, the G′ value recovered to the initial G′ value for the dynamic hydrogels in 3 min. Meanwhile, in the two consecutive cycles we evaluated the dynamic hydrogel showed consistent recovery behavior.

The injectability of the dynamic hydrogel was shown by the drastic decrease in the viscosity when the external shear rate was increased (Figure 2D). The viscosity decreased from 220 Pa·s at the shear rate of 1.25 s^−1^ and substantially reduced to 6.2 Pa·s at the rate of 100 s^−1^, suggesting a shear-thinning property of the dynamic hydrogel.

**Figure 3:**
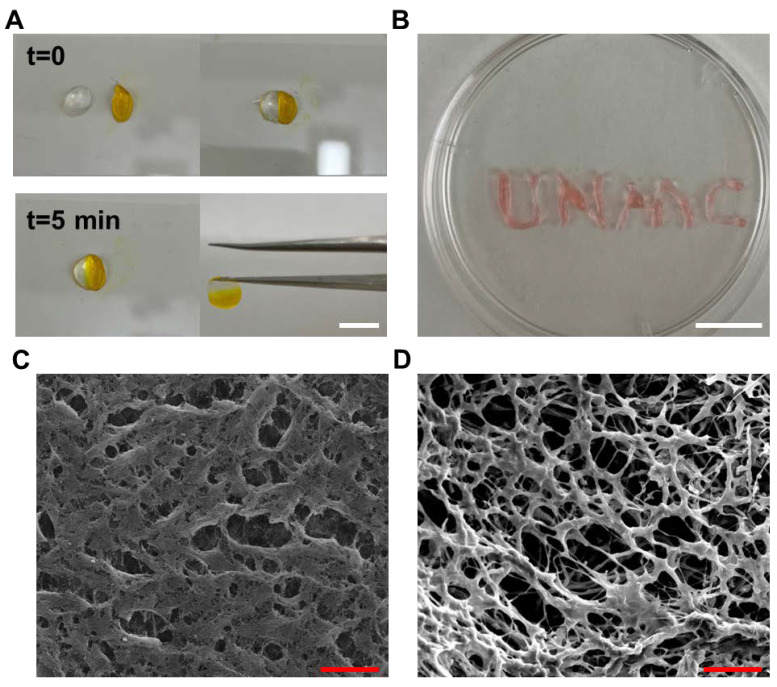
Macro- and micro-scopical characterization of Alg-PBA hydrogels. (**A**) Illustration of self-healing property of dynamic hydrogel; (**B**) Syringe injectability of Alg-PBA-PVA hydrogel; (**C**,**D**) SEM images of dynamic hydrogel before (**C**) and after (**D**) CaCl_2_ crosslinking. Scale bar (white) 5 mm, scale bar (red) 25 µm.

To better demonstrate the self-healing property of Alg-PBA-PVA, a blank hydrogel was placed next to a yellow dye-colored hydrogel, allowing two pieces to heal with each other at room temperature (Figure 3A). It was found that the boundary between the contacting hydrogels quickly disappeared. The yellow dye from the other piece of hydrogel started diffusing into the blank hydrogel. The two pieces of hydrogel were healed into one single piece in 5 min and healed hydrogel could be easily held up by griping one end of the hydrogel.

The injectability of the Alg-PBA-PVA hydrogel was also examined macroscopically. The dynamic hydrogel was transferred into an insulin syringe and was fluidly extruded through a 30 G needle, behaving like the fluid after pushing the plunger (Figure 3B). When the hydrogel came out of the needle, it promptly returned to the hydrogel state. These results, together with the rheological results, indicated satisfying self-healing and shear-thinning properties of the Alg-PBA-PVA dynamic hydrogel. The behavior of the alginate dynamic hydrogel was similar to many reported boronate ester bond-based dynamic hydrogels.

The hydrogels were examined microscopically by SEM and both showed microporous structures (Figure 3C,D), albeit distinguished from each other. Compared to the non-crosslinked hydrogel, the crosslinked hydrogel showed more pores in the 10–30 µm size range and more uniform fibers around the pores.

In the end, we characterized the stability of the Alg-PBA hydrogels (Figure S2). The dynamic Alg-PBA-PVA hydrogel was unstable in the cell growth medium at 37 °C, with 50% of the initial weight lost after 1 day, which made it unsuitable for cell culture purpose and in vivo application. The poor stability was attributed to the rich amount of glucose; a diol-rich small molecule in the medium could compete with PVA and form boronate ester complex with Alg-PBA [31]. The consequence was the fast degradation of the dynamic hydrogel network. However, the Alg-PBA-Ca hydrogel maintained over 50% of their initial weights after 14 days, indicating enhanced stability of Alg-PBA hydrogel after secondary crosslinking. The degradation profile was similar to that of the traditional Ca^2+^ crosslinked alginate hydrogel. Secondary ionic crosslinking provided an easy but effective method to improve the structural integrity of the dynamic alginate hydrogel. This method is different to our previous chemical crosslinking approaches, which relied on the introduction of a secondary functional group to the polysaccharide as well as another functionalized polymer conjugate as a crosslinker.

### 2.2. Cytocompatibility of Alg-PBA Hydrogels

**Figure 4:**
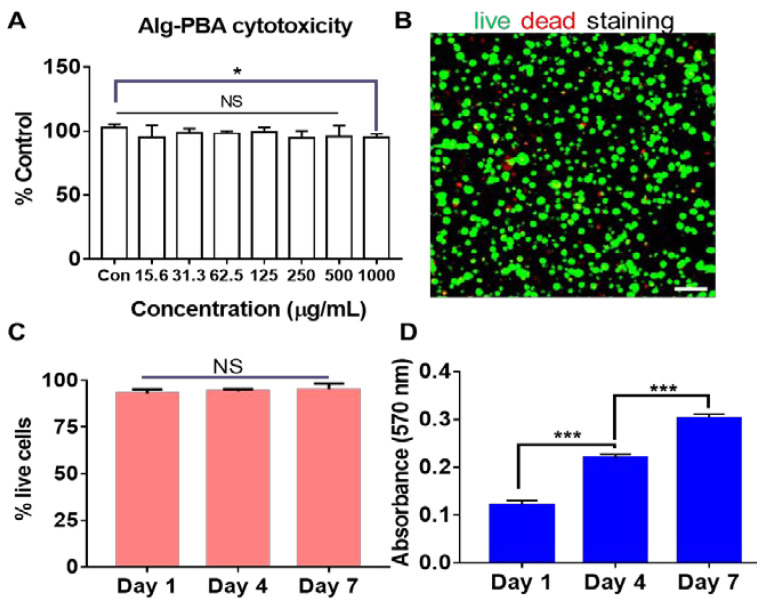
Cytocompatibility of Alg-PBA dynamic hydrogel precursor and dynamic alginate hydrogels. (**A**) Cytotoxicity of synthesized Alg-PBA conjugate towards L929 cell determined by MTT assay. (**B**) Representative live and dead staining of L929 cells in stabilized hydrogel at day 7. (**C**) Viability of L929 encapsulated in hydrogel from day 1 to day 7. (**D**) Cell proliferation of L929 cells in hydrogel from day 1 to day 7. Scale bar = 100 µm. NS: not significant; * *p* < 0.05; *** *p* < 0.001.

The cytotoxicity of the synthesized hydrogel precursor was first evaluated on mouse fibroblast cell line L929. PVA was a well-known cyto-compatible biomaterial and was not studied. At 24 h, no significant difference in terms of the cell density was observed in the treatment groups from 15 to 500 µg/mL of the conjugate concentration. However, at 1000 µg/mL concentration the treated cells showed a slightly lower (on average 8%, *p* < 0.05) cell density than the control (Figure 4A), implying minimal effect of the conjugate on the growth of the fibroblasts.

The influence of the crosslinked dynamic hydrogel on the viability and proliferation of encapsulated L929 cells was evaluated by live and dead staining and the MTT assay at each time point. After mixing the precursors, the hydrogel contained around 1.9% Alg-PBA and 1% PVA. The L929 showed over 90% viability in the hydrogels from day 1 to day 7, as shown by the live/dead assay (Figure 4B,C); no significant decrease in cell viability was found at three time points (Figure 4C). L929 cells proliferated well inside the crosslinked dynamic hydrogel during the 7-day culture period (Figure 4D). Based on the absorbance value, the cell number almost doubled at day 4 compared to day 1. On day 7, the cell number was nearly three times higher than that of day 1. Given the apparent degradation of the hydrogel (~25%) on day 7, the cells should proliferate faster than our measured result. In summary, all results suggested good cytocompatibility of Alg-PBA hydrogels towards mammalian cells.

### 2.3. Assessment of Alg-PBA Hydrogels Based Bioinks for 3D Bioprinting

The viscosity of bioink for 3D printing had a great impact on achieving successful fabrication of scaffolds with the desired structure. Non-modified alginate solutions suffer from low viscosity and are not suitable as bioinks unless at high concentration or mixed with other viscosity-modifying agents. Inspired by the shear-thinning behavior of the dynamic alginate hydrogel (Alg-PBA-PVA) as well as the stability and cyto-compatibility of the crosslinked hydrogel, we sought to apply the dynamic alginate hydrogel as a bioink for 3D bioprinting. Under the optimized printing pressure, the thickness of the extruded filaments was dependent on the diameter of the printing nozzle. We extruded the dynamic alginate hydrogel through the 22 G and 25 G nozzles and observed continuous printed strand with consistent thickness from both nozzles (Figure S3). The thickness of the printed filament was 700 µm on average when using the 22 G nozzle and 300 µm on average when using the 25G nozzle.

**Figure 5:**
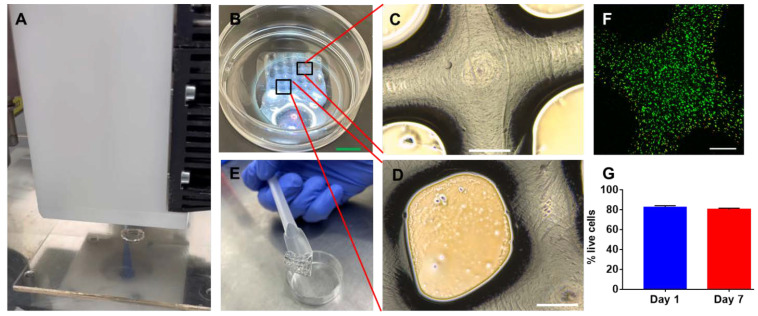
A 3D bioprinting study using mouse chondrocyte laden dynamic alginate hydrogels. (**A**) Illustration of extrusion based bioprinting process using dynamic hydrogel as a bioink to fabricate a cylinder shape scaffold; (**B**) top view image of a bioprinted grid scaffold; (**C**,**D**) micro-scopic image of the bioprinted scaffold with a grid structure. Black box indicates the magnified area; (**E**) representative image of a bioprinted scaffold after CaCl_2_ crosslinking lifted by a spatula; (**F**) live and dead staining of mouse chondrocytes in the printed grid scaffold after printing. (**G**) Viability of the mouse chondrocyte in the printed scaffold at day 1 and day 7. Scale bar (green) = 5 mm, scale bar (white) = 500 µm.

To verify the printing fidelity of the dynamic alginate hydrogel, constructs with cylindrical and grid structures were printed (Figure 5A,B) using the 22 G nozzle. For both constructs, the printed scaffolds presented high structural similarity to the 3D design models. At least six layers could be easily printed for each scaffold. For the grid scaffold, the angular pore shape was well retained (Figure 5D). The printed scaffolds were further stabilized by the CaCl_2_ solution immersion. After crosslinking, the stabilized scaffolds could be easily lifted and transferred into cell culture medium using the spatula (Figure 5E). Additionally, the printing process had minimal influence on the cell viability (>85%) determined from the live and dead staining as shown in Figure 5F. The fluorescent region correlated well with the cross-section area of the printed strand. More dead cells were located in the periphery of the strands, which might be caused by shearing stress during the extrusion. The primary mouse chondrocytes maintained good viability in the bioprinted scaffolds. Our study revealed the viability of the chondrocytes was ~83% on average in the scaffold at day 1; viability had barely decreased to ~80% at day 7 (Figure 5G), which is comparable to previous reported bioinks [32].

### 2.4. Antioxidative Property of Alg-PBA Hydrogels

**Figure 6:**
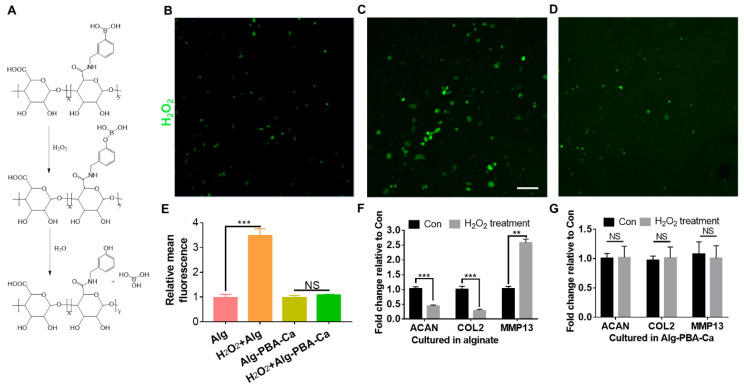
Characterization of anti-ROS property of the dynamic alginate hydrogel. (**A**) Mechanism behind the H_2_O_2_ scavenging property of Alg-PBA. (**B**–**D**) ROS fluorescent staining of mouse chondrocytes before H_2_O_2_ exposure in the alginate hydrogel (**B**), after H_2_O_2_ treatment in the alginate hydrogel (**C**), and with H_2_O_2_ exposure in bioprinted Alg-PBA-Ca hydrogel (**D**). (**E**) Quantitative measurement of the intracellular ROS levels in different groups. (**F**) Mouse chondrocytes showed significant downregulation of two ECM anabolic genes (ACAN and COL2) and upregulation of one catabolic gene (MMP13) when cultured in alginate hydrogel after H_2_O_2_ exposure. (**G**) Mouse chondrocyte showed no statistical difference in gene expression in three ECM genes when cultured in the bioprinted Alg-PBA-Ca hydrogel after H_2_O_2_ exposure. Scale bar = 100 µm. NS: not significant; ** *p* < 0.01; *** *p* < 0.001.

To determine whether the hydrogel could suppress the oxidative stress on cells, mouse chondrocytes embedded in the traditional calcium crosslinked alginate hydrogel or the bioprinted Alg-PBA-Ca hydrogel were exposed to H_2_O_2_ stimulus in the culture medium to mimic oxidative stress. The intracellular oxidative stress was stained by an H_2_O_2_ responsive probe known as H_2_DCFDA. H_2_DCFDA is a cell permeable and non-fluorescent molecule; however, it becomes fluorescent after activation by the intracellular H_2_O_2_ [33]. As shown in Figure 6B–D, there were more fluorescent cells in the alginate hydrogel after H_2_O_2_ treatment than in both the alginate hydrogel group without H_2_O_2_ treatment and the Alg-PBA-Ca group after H_2_O_2_ treatment. At the same time, the alginate hydrogel group after H_2_O_2_ treatment showed almost 3.5 times increase in the fluorescence intensity compared to the alginate group without H_2_O_2_ treatment (*p* < 0.001, Figure 6E). On the contrary, there is no statistical difference regarding the fluorescence intensity in the Alg-PBA-Ca hydrogel group before and after H_2_O_2_ treatment. The intensity in the Alg-PBA-Ca hydrogel group after H_2_O_2_ treatment was much lower than the alginate hydrogel group after H_2_O_2_ treatment, demonstrating the Alg-PBA-Ca hydrogel efficiently scavenge the H_2_O_2_ in the medium and mitigate the oxidative stress on the chondrocytes at least in vitro. The effect was considered as resulting from a reaction of the boronic acid/boronate ester with H_2_O_2_, generating the low cytotoxic boric acid after the reaction (Figure 6A).

To assess the biological influence or the value of the antioxidative hydrogel on chondrocytes, the effect of excess H_2_O_2_ on the regulation of two ECM anabolic genes (i.e., ACAN and COL2) as well as one catabolic gene (i.e., MMP13) were evaluated by RT-PCR. As shown in Figure 6F,G, mouse chondrocytes encapsulated in the traditional alginate hydrogel with no H_2_O_2_ scavenging ability, showed an average of ~60% and 75% (*p* < 0.001) reduction in ACAN and COL2 expression, respectively, after H_2_O_2_ exposure. However, the chondrocytes in the Alg-PBA-Ca bioink maintained the same level of expression of ACAN and COL2 in the presence of H_2_O_2_. Meanwhile, the expression of MMP13, a critical catabolic factor responsible for the degradation of type II collagen in the cartilage ECM [34], increased by over 2.5-fold (*p* < 0.01) after H_2_O_2_ exposure in chondrocytes in the alginate hydrogel. In contrast, there was no significant difference regarding the MMP13 expression when the chondrocytes embedded in the Alg-PBA bioink were exposed to H_2_O_2_ in vitro. The downregulation of ACAN and COL2 genes and upregulation of MMP-13 gene for chondrocytes in the alginate hydrogel after H_2_O_2_ exposure from our result is consistent with the previous study conducted on 2D cultured chondrocytes exposed to H_2_O_2_ [35]. It reproduced the transcriptional changes of chondrocytes under the pathological oxidative stress during the osteoarthritis progression [36]. Due to the H_2_O_2_ scavenging ability of the boronate ester, many boronate ester-derived small molecules and nanoparticles were developed with antioxidative properties and, thus, anti-inflammation therapeutic potentials [37,38,39]. Our study delineated such efficacy of the Alg-PBA bioink as the bioprinted hydrogel significantly reduced the intracellular oxidative stress and protected the loaded chondrocytes from H_2_O_2_-induced transcriptional changes of three genes (ACAN, COL2, and MMP13), which are important for the functions of chondrocytes. Previous results also suggested that scavenging of H_2_O_2_ by the boronate ester could suppress the production of some pro-inflammatory factors, such as tumor necrosis factor (TNF)-α and interleukin (IL)-1β, from macrophages under oxidative stress [37]. The potential anti-inflammatory effects in macrophages could be another incentive to apply the bioprinted dynamic alginate hydrogel as a cartilage tissue engineering scaffold and will be evaluated in a future study.

## 3. Conclusions

In summary, our synthesized PBA grafted alginate conjugate was used to readily prepare the dynamic alginate hydrogel with good self-healing and shear-thinning properties. The dynamic alginate hydrogel could be easily stabilized with the ionic crosslinking method and was cyto-compatible. For the first time, the dynamic alginate hydrogel was applied as a bioink for 3D bioprinting. Scaffolds fabricated with the dynamic alginate bioink showed good structural fidelity and supported the growth of embedded primary mouse chondrocytes for at least 7 days. Moreover, the bioink reduced the ROS stress of the encapsulated chondrocytes in vitro and protected the chondrocytes from H_2_O_2_-induced phenotype change. The versatility of this novel bioink indicates its great potential to print constructs with the antioxidative ability to enhance cartilage tissue regeneration.

## 4. Materials and Methods

### 4.1. Synthesis of Alginate-Phenylboronic Acid (Alg-PBA) Precursor

The Alg-PBA conjugate was synthesized after conjugating the 3-aminomethylphenylboronic acid (PBA, Alfar Aesar, Ward Hill, MA, USA) to alginate backbone using 4-(4,6-dimethoxy-1,3,5-triazin-2-yl)-4-methymorpholinium chloride (DMTMM, TCI America, Portland, OR, USA) as the coupling agent following a modified protocol from a previous study [18]. In general, 100 mg alginate (0.5 mmol, Protanal^®^ LF 10/60, FMC Corporation, Philadelphia, PA, USA) was dissolved in 10 mL de-ionized (DI) water. A total of 23.5 mg PBA (0.125 mmol) and 37.5 mg DMTMM (0.125 mmol), respectively, were added to the alginate solution. After complete dissolution, the pH of the solution was adjusted to 6.5 with 1 M HCl (Fisher Chemical, Hampton, VA, USA) solution. To tune the conjugation ratio, another reaction was conducted using 0.5 mmol alginate, 0.25 mmol PBA, and 0.25 mmol DMTMM at pH 6.5 in 10 mL water. Each reaction mixture was stirred at room temperature for 3 days before being transferred to a 6–8 kDa molecular weight cut-off (MWCO) dialysis bag (Spectrum). The mixture was dialyzed against DI water for 3 days, with the water changed thrice every day at room temperature. The purified conjugate was lyophilized and stored in the freezer before use.

### 4.2. Proton Nuclear Magnetic Resonance (^1^H NMR) Analysis of Alg-PBA

^1^H NMR spectrum was collected on a 500 MHz Bruker NMR system and analyzed using Topspin 4.0 software. Each polymer conjugate was dissolved in D_2_O (Acros Organics, Morris Plains, NJ, USA) at 6 mg/mL for NMR acquisition and the chemical shifts were referred to the solvent peak of D_2_O at 4.78 ppm at 25 °C during data analysis. To figure out the grafting ratio of phenylboronic acid, 25 mM dimethylformamide (DMF) was added into the polymer conjugate solution in D_2_O as a reference material; it was then calculated based on the ratio of the integral of aromatic protons from the conjugated phenylboronic acid group (7.3~7.7 ppm, -C_6_*H*_4_) to the integral of the DMF formamide proton peak (at 7.8 ppm, -C*H*O), as well as the existing amount of DMF and Alg-PBA in D_2_O as described in a previous study.

### 4.3. Preparation of Alg-PBA Hydrogels

The Alg-PBA dynamic hydrogel was prepared by mixing 2.5 wt% of Alg-PBA, dissolved in sodium chloride (NaCl) buffer (0.15 M, Fisher Chemical) at pH 7 ± 0.2, with 4 wt% PVA (13 kDa, Acros Organics), dissolved in NaCl at pH 7 ± 0.2, in a 3:1 volume ratio at room temperature. The hydrogel gelated quickly by complexation formation between the PBA groups in Alg-PBA and 1,3-diol groups in PVA (the boronate ester dynamic bond). The prepared dynamic hydrogel was referred to as the Alg-PBA-PVA. To stabilize the dynamic hydrogel, Alg-PBA-PVA was immersed in a 50 mM calcium chloride (CaCl_2_) solution in NaCl buffer for 10 min at room temperature; the Ca^2+^ crosslinked dynamic hydrogel was referred to as the Alg-PBA-Ca.

### 4.4. Rheological, Self-Healing and Injectability Studies of Alg-PBA Hydrogels

The Discovery HR-2 rheometer (TA Instruments, New Casstle, DE, USA) was used to characterize the rheological properties of Alg-PBA-PVA and Alg-PBA-Ca hydrogels at 37 °C with four testing protocols. For the Alg-PBA-PVA hydrogel, 150 µL of 2.5% Alg-PBA solution was first transferred into the 20 mm parallel plate; 50 µL of 4% PVA solution was quickly mixed with the Alg-PBA solution. The geometry gap was then set at 500 µm and the strain was set at 10%. Time-sweep study was evaluated at the frequency of 2π rad/s to monitor the storage modulus (G′) and loss modulus (G″) change over time and follow the gelation kinetics of the dynamic hydrogel. The frequency sweep measurement was then conducted, with the angular frequency varying from 0.63 to 62.8 rad/s. The self-healing property of the dynamic hydrogel was then determined by an alternate step strain sweep test under a constant frequency (2π rad/s). The amplitude oscillatory strains were shifted from a small strain (γ = 10%) to a successive large strain (γ = 500%) within the 240 s cycle time; 2 cycles were recorded. In the end, to evaluate the shear-thinning behavior, flow sweep studies were tested; the shear rate linearly ramped up from 1 to 100 s^–1^ and the viscosity was documented at different shear rates. For the Alg-PBA-Ca hydrogel, 200 µL of the crosslinked hydrogel was crosslinked in a 20 mm diameter mold and quickly placed between the 20 mm parallel plates after crosslinking. The time-sweep and frequency-sweep studies were performed at the frequency of 2π rad/s and with 10% strain; the geometry gap was set at 500 µm.

To investigate the self-healing property, one piece of Alg-PBA-PVA hydrogel was prepared by mixing 30 µL of 2.5% Alg-PBA with 10 µL of 4% PVA; another piece of Alg-PBA-PVA hydrogel was prepared with the same composition with additional 1 µL of yellow-colored food dye solution. After gelation, two pieces of hydrogels were placed next to each other in a coverslip glass. The glass was covered within a dish and kept at room temperature to allow the occurrence of the healing process of two hydrogels. After 5 min, the healed hydrogel was lifted with a tweezer for observation.

Additional 150 µL solutions of 2.5% Alg-PBA, 50 µL 4% PVA, and 5 µL of red colored dye were mixed to prepare the hydrogel for the injectability demonstration. After gelation, the hydrogel was loaded into a 30-gauge (G) needle-capped insulin syringe and the hydrogel was extruded through the needle into a dish after manually pushing the plunger.

### 4.5. Scanning Electron Microscope (SEM) Study of Alg-PBA Hydrogels

Alg-PBA hydrogels before and after CaCl_2_ crosslinking were freeze-dried completely after preparation. The surfaces of the cross-section of two different hydrogels were sprayed with thin layers of gold. The morphology and pore sizes of all samples were imaged using a scanning electron microscope (SEM, FEI Quanta 200, Hillsboro, OR, USA).

### 4.6. The Cytocompatibility of the Hydrogel Precursor and Prepared Hydrogels

Mouse fibroblast cell L929 were grown and expanded in the cell growth medium containing 10% fetal bovine serum (FBS, Gibco, Waltham, MA, USA) and 1% Penicillin-Streptomycin (P/S, Invitrogen, Waltham, MA, USA); during this task, we used Dulbecco’s Modified Eagle medium with Nutrient Mixture F-12 (DMEM/F-12, Gibco) at 37 °C in a humidified incubator (Thermo, Waltham, MA, USA) containing 5% CO_2_.

For the cytotoxicity study, 1 × 10^4^/well L929 were seeded in each well of a 48-well plate and the cells were attached overnight. On the next day, the UV-sterilized Alg-PBA conjugate at different concentrations between 15.6 µg/mL to 1000 µg/mL was prepared in cell growth medium. The old medium in each cell-attached well was removed and replaced with the medium containing the polymer conjugate, followed by 24 h incubation. The Control Group was cultured in the fresh growth medium. The cytotoxicity was assessed by an MTT ([3-(4,5-dimethylthiazol-2-yl)-2,5-diphenyltetrazolium bromide, Sigma, St. Louis, MO, USA) assay [40,41]. Generally, the media containing Alg-PBA was aspirated, fresh culture media containing 0.5 mg/mL MTT was added, and the cells were incubated for another 3 h. The media was then emptied and 0.5 mL DMSO (Fisher Chemical) was added to each well. The culture plate was rotated for 30 min to completely dissolve the dye. We then transferred 0.1 mL of DMSO from each well to one well of a 96-well plate; the absorbance was determined at 540 nm wavelength with the microplate reader (Biotek, Winnooski, VT, USA). The absorbance value of each well was normalized to the average absorbance value in the Control Group. Each different group had 4 replicates.

For the hydrogel cytocompatibility study, L929 cells were first suspended in the UV-irradiated Alg-PBA precursor solution at 1 × 10^6^/mL in DMEM/F-12 medium. The PVA was sterile filtered. The cell-laden hydrogel was gelated after the PVA solution addition and then stabilized with calcium crosslinking. Each L929 encapsulated hydrogel was individually transferred to a corresponding well of a 12-well plate; 2 mL of cell culture medium was then added. The cells inside hydrogels were cultured at 37 °C in the incubator. The medium was changed every two days. At each time point until day 7, live/dead fluorescent staining was used to quantify cell viabilities inside hydrogels [42]. Each group had 4 replicates. At the same time, another four pieces of the hydrogels were collected to evaluate the cell proliferation by the MTT assay, as mentioned above.

### 4.7. Isolation, Culture, and 3D Bioprinting of Mouse Chondrocytes

Mouse chondrocytes collected from cartilage tissues of embryonic day 17.5 wildtype mice from Acan^cmd^/NKruJ matings (Stock #010522; Jackson Laboratories, Bar Harbor, ME, USA) by our collaboration lab were given to us for free [22]. All procedures were approved by the Institutional Animal Care and Use Committee (IACUC) at UNMC. The hind limb growth plates were biopsied and digested in 1% (*w*/*v*) pronase (Sigma) for 30 min; this was followed by digestion with 0.25% (*w*/*v*) collagenase (Sigma) for 3 h at 37 °C to remove all extracellular matrix from the cartilage. Chondrocytes were centrifuged at 150× *g* for 5 min and resuspended with the growth medium. The growth medium contained the MEM alpha medium without phenol red (Gibco) supplemented with 10 mM β-glycerophosphate (Sigma), 50 µg/mL L-ascorbic acid (Sigma), 1 nM dexamethasone (Sigma), 1 mg/mL proline (Sigma), 1% antioxidant (Sigma), 1 mM sodium pyruvate (Gibco), 1% nonessential amino acid (Gibco), and 1% Insulin–Transferrin–Selenium +3 media supplement (ITS +3, Sigma) [43].

To prepare mouse chondrocyte-laden bioink, 2.5 × 10^6^ chondrocytes were first suspended in 1 mL of 2.5% Alg-PBA precursor solution in DMEM/F-12, followed by mixing with 4% PVA solution. The bioink was transferred into the printing syringe (Nordson EFD, Westlake, OH, USA) and loaded into the 3D printer. A Bioplotter 3D (EnvisionTEC, Dearborn, MI, USA) extrusion printer was used and the syringe was capped with printing nozzles containing 22 G or 25 G conical tips. The printing parameters and patterns were controlled by VisualMachines V 2.8.129r1 software (EnvisionTEC). Each layer of the cylindrical construct was made of a circle with a diameter of 8 mm. The grid construct had a square shape filled with strands in each layer; the grid construct had a side length of 1.5 cm, while the distance between adjacent strands was ~2.5 mm. The printing pressure for the bioink was 2.00 ± 0.3 bar. The typical printing speed was 8 mm/s.

### 4.8. H_2_O_2_ Treatment and ROS Responsive Fluorescent Staining

The bioprinted and crosslinked Alg-PBA-Ca hydrogel were treated with H_2_O_2_ to mimic the oxidative stress. A 2% alginate hydrogel encapsulated with 2 × 10^6^/mL chondrocytes and crosslinked with 50 mM CaCl_2_ was used as the control. The chondrocytes were cultured in the growth medium overnight after bioprinting. Before H_2_O_2_ treatment, each piece (40 µL) of chondrocyte-laden alginate or Alg-PBA-Ca hydrogel was put into one well of the 24-well plate and immersed in DMEM/F-12 for 1 h to wash the residual cell culture medium inside the gels. After emptying the wash medium, another 1 mL of fresh DMEM/F-12 containing 500 µM H_2_O_2_ was added to every well; the chondrocyte-laden alginate or Alg-PBA-Ca hydrogel were then cultured for another 24 h at 37 °C.

To investigate the in vitro ROS (H_2_O_2_) scavenging ability of the Alg-PBA-Ca hydrogel, the H_2_O_2_ responsive fluorescent probe, 2′,7′-dicholorodihydro fluorescein diacetate (H_2_DCFDA, Millipore), was used to measure the intracellular H_2_O_2_ levels after H_2_O_2_ exposure [18]. H_2_DCFDA probe was prepared at 5 µM in DMEM/F-12 as a working solution. Chondrocytes inside the alginate or Alg-PBA-Ca hydrogel after 2 h treatment of 500 µM H_2_O_2_ were taken out of the medium, washed twice with DMEM/F-12, and stained with the probe solution (0.5 mL) for 30 min. After emptying the probe solution, the fluorescence of the cells inside hydrogels was imaged by the confocal microscopy (Zeiss 880, Zeiss, Oberkochen, Germany) with the same parameters for all groups. For the Control Group, chondrocytes inside hydrogels were cultured in the DMEM/F-12 without H_2_O_2_ treatment; however, they were incubated with the probe solution following the same procedure at the same time. The intracellular fluorescence intensity was determined using a previously reported method that involved using the ImageJ 1.53 [44] software to subtract the mean fluorescence intensity of the region of interest (or fluorescent cells) from the fluorescence intensity of the dark background.

### 4.9. RNA Isolation and Quantitative Real Time Polymerase Chain Reaction (RT-PCR)

Bioprinted Alg-PBA-Ca hydrogel and alginate hydrogel encapsulated with mouse chondrocytes were first depolymerized by a depolymerizing solution (Cell Applications Inc., San Diego, CA, USA) to remove Ca^2+^ crosslinking; this was followed by homogenization with a bead mill homogenizer (Fisher Scientific, Waltham, MA, USA) in the cell lysis buffer (Qiagen, Hilden, Germany) to release the cytoplasm. The total RNA was extracted from the cell lysate with the RNeasy mini-kits (Qiagen) following the protocol. The extracted total RNA was transcribed into complementary DNA (cDNA) with an iScript cDNA synthesis kit (Quantabio, Berverly, MA, USA). Real-time PCR analysis was conducted in a StepOnePlus™ Real-Time PCR System (Thermo Scientific, Waltham, MA, USA) with SYBR Green Supermix (Bio-Rad, Hercules, CA, USA) dye. The cDNA samples were evaluated for the target genes and the housekeeping gene (Actb). The primer sequences are shown in Table S1. The relative expression of each studied gene was determined by the comparative Ct (2 ^−ΔΔCt^) method [45]. Each group had three replicates.

### 4.10. Statistical Analysis

Quantitative data are represented as the mean ± standard deviation (SD). The statistical analysis was performed with an unpaired student *t*-test by the GraphPad Prism 7. The significance was presented as follows: * *p* < 0.05; ** *p* < 0.01; *** *p* < 0.001.

## Data Availability

Data is contained within the article.

## Supplementary Materials

The following supporting information can be downloaded at: https://www.mdpi.com/article/10.3390/gels9040312/s1; Figure S1: The grafting ratio of PBA in alginate can be determined by ^1^H NMR in D_2_O with the addition of 25 mM DMF; Figure S2: Stability of Alg-PBA hydrogel before and after calcium crosslinking in the cell culture medium (*n* = 3); Figure S3: Influence of printing nozzle diameter on the fiber thickness of the printed grid scaffold. Scale bar 2.5 mm; Table S1: Primer sequence for the RT-PCR study.
