# Supplementary material for: Dynamic Alginate Hydrogel as an Antioxidative Bioink for Bioprinting
Source: Gels. 2023 Apr 7;9(4):312. doi: 10.3390/gels9040312 (PMC10137987; doi:10.3390/gels9040312)
Supplement: Supplementary file 1 [file gels-09-00312-s001.zip › gels-2313955-supplementary.pdf]

Dynamic Alginate Hydrogel as an Antioxidative Bioink for Bioprinting

Wenhai Zhang [1,2], Mitchell Kuss [2], Yi Yan [3,*] and Wen Shi [2,*]

1 First Hip Department of Orthopedics, Tianjin Hospital, Tianjin 300211, China

2 Mary & Dick Holland Regenerative Medicine Program, University of Nebraska Medical Center, Omaha, NE 68198, USA

3 Healthcare Security Office, Union Hospital, Tongji Medical College, Huazhong University of Science and Technology, Wuhan 430074, China

* Correspondence: ezhouyanyi@163.com (Y.Y.); donatellowenshi@gmail.com (W.S.)

Table S1: Primer sequence for the RT-PCR study.

Gene	Genbank ID	Forward sequence	Reverse sequence	Product size
Acan	NM_007424.3	TCACTGTTACCGCCACTTTCC	TGCTGCTCAGATGTGACTGC	86
Col2a1	NM_031163.3	CGAGTGGAAGAGCGGAGACTAC	CCAGTTTTTCCGAGGGACAGT	136
Mmp13	NM_008607.2	GGTTATGACATTCTGGAAGGTTATCC	CGTGGTTCTCAGAGAAGAAGAGG	130
Actb	NM_007393.5	AAATCGTGCGTGACATCAAAGA	GCCATCTCCTGCTCGAAGTC	64

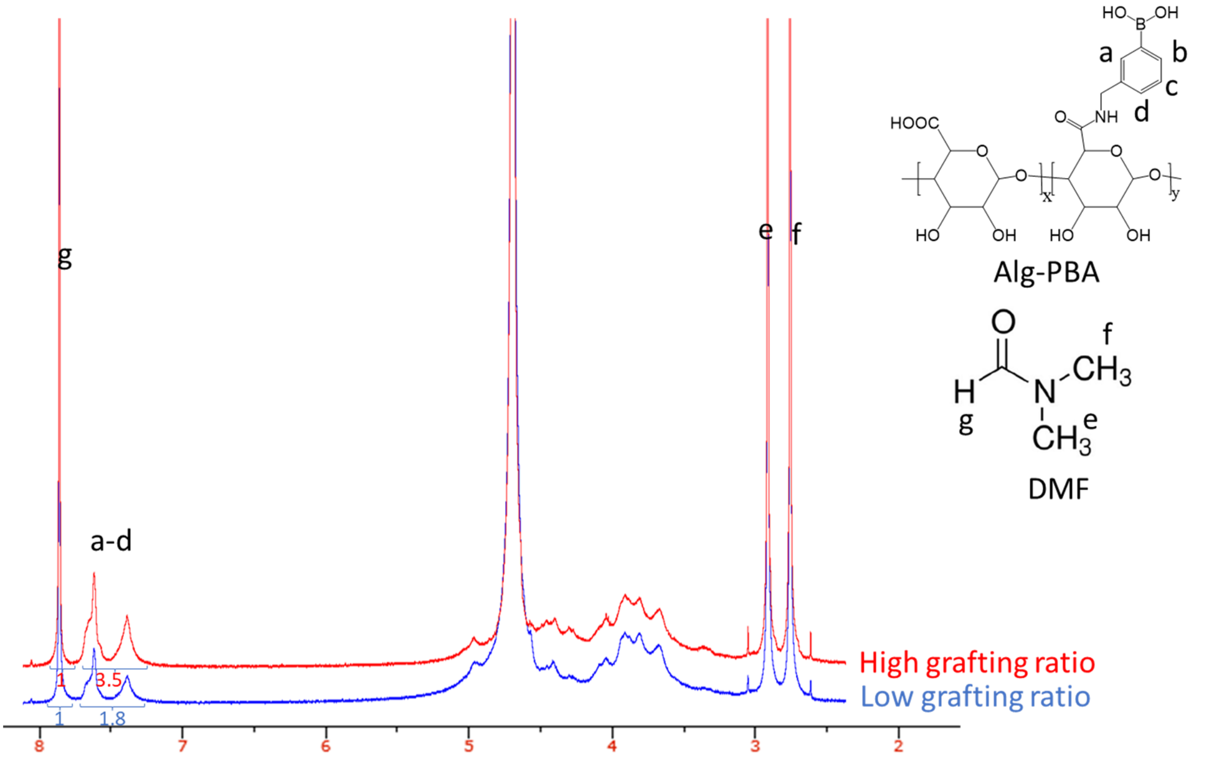

Figure S1: The grafting ratio of PBA in alginate can be determined by ^{1}H NMR in D_2O with the addition of 25 mM DMF.

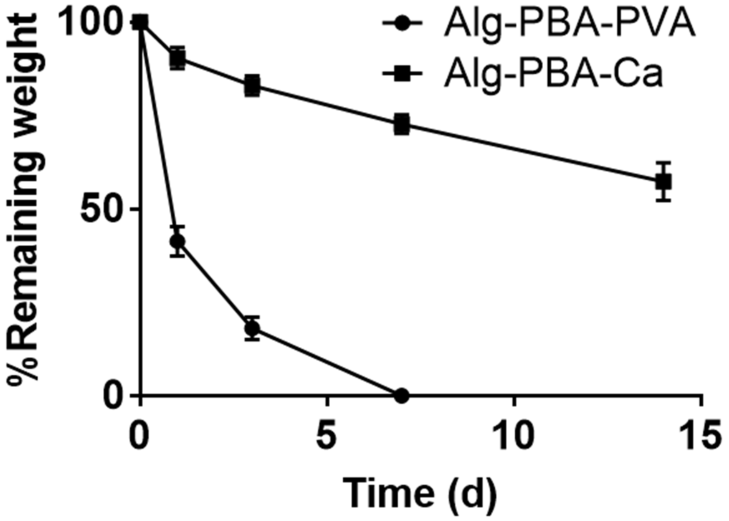

Figure S2. Stability of Alg-PBA hydrogel before and after calcium crosslinking in the cell culture medium.

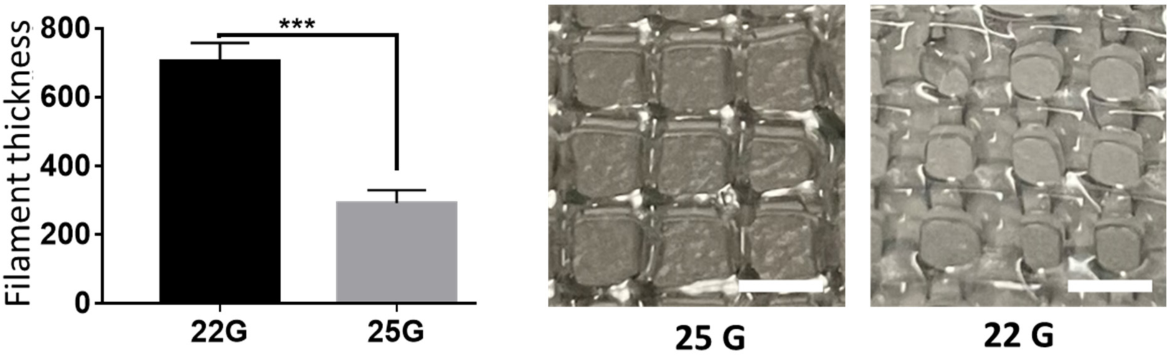

Figure S3. Influence of printing nozzle diameter on the filament thickness of the printed grid scaffold. Scale bar 2.5 mm. *** $p < 0.001$.
